# Mechanism of lead adsorption by a *Bacillus cereus* strain with indole-3-acetic acid secretion and inorganic phosphorus dissolution functions

**DOI:** 10.1186/s12866-023-02795-z

**Published:** 2023-03-04

**Authors:** Qingrong Li, Wenbo Zhang, Sentai Liao, Dongxu Xing, Yang Xiao, Donglai Zhou, Qiong Yang

**Affiliations:** 1 Sericultural and Agri-Food Research Institute, Guangdong Academy of Agricultural Sciences, Guangzhou, 510610 China; 2 Key Laboratory of Urban Agriculture in South China, Ministry of Agriculture and Rural Affairs, GuangZhou, 510610 China

**Keywords:** Plant growth promoting, Heavy metal, Lead, Microbial remediation

## Abstract

**Background:**

Heavy metal pollution has become a major source of environmental pollution because of increasing industrialization. Microbial remediation is a promising approach to remediate lead-contaminated environments owing to its cost-effective, environment-friendly, ecologically sustainable, and highly efficient properties. In this study, the growth-promoting functions and lead-adsorption ability of *Bacillus cereus* SEM-15 were examined, and the functional mechanism of the strain was preliminarily identified using scanning electron microscopy, energy spectrum, infrared spectrum, and genome analyses, providing theoretical support for utilization of *B. cereus* SEM-15 in heavy metals remediation.

**Results:**

*B. cereus* SEM-15 showed strong ability to dissolve inorganic phosphorus and secrete indole-3-acetic acid. The lead adsorption efficiency of the strain at lead ion concentration of 150 mg/L was more than 93%. Single factor analysis revealed the optimal conditions for heavy metal adsorption by *B. cereus* SEM-15 (adsorption time, initial lead ion concentration, pH, and inoculum amount were 10 min, 50–150 mg/L, 6–7, and 5 g/L, respectively) in nutrient-free environment, with the lead adsorption rate reaching 96.58%. Scanning electron microscopy of *B. cereus* SEM-15 cells before and after lead adsorption showed adherence of a large number of granular precipitates to the cell surface after lead adsorption. X-Ray photoelectron spectroscopy and Fourier transform infrared spectroscopy results indicated the characteristic peaks of Pb–O, Pb–O-R (R = functional group), and Pb–S bonds after lead adsorption, and a shift in the characteristic peaks of bonds and groups related to C, N, and O. Genome annotation results showed the presence of genes related to heavy metals tolerance and plant growth promotion in *B. cereus* SEM-15, providing a molecular basis for the strain’s heavy metals tolerance and plant growth promotion functions.

**Conclusions:**

This study analyzed the lead adsorption characteristics of *B. cereus* SEM-15 and the associated influencing factors, and discussed the adsorption mechanism and related functional genes, providing a basis for clarifying the underlying molecular mechanism and offering a reference for further research on plant-microorganisms combined remediation of heavy metals polluted environments.

**Supplementary Information:**

The online version contains supplementary material available at 10.1186/s12866-023-02795-z.

## Background

With the development of industrialization, heavy metals pollution in urban soil, farmland soil, water bodies, and even the atmosphere has gradually become one of the main types of environmental pollution [1–4]. Heavy metals, such as lead, can quickly accumulate through food chains, seriously threatening the health of animals and humans [5, 6]. Methods for remediation of heavy metals pollution mainly include physical, chemical, and biological methods [7–10]. When compared with traditional physical and chemical treatments, microbial remediation strategy is a promising alternative to remediate lead-contaminated environments owing to its cost-effective and environment-friendly properties [11], and it has gradually become a widely used approach in recent years [12].

Various microorganisms, such as bacteria, fungi, and algae, have been shown to exhibit high tolerance to lead and excellent lead immobilization capacity [13–16]. Chen et al. [17] found that the Pb^2+^ removal rate in the fixed solution inoculated with *Enterobacter* sp. was 60.85% higher than that in biochar alone. Although many bacterial strains have been reported to have good heavy metals tolerance and passivation repair function, the demand for microbial resources that can improve the growth and yield of plants in heavy metals contaminated soil has been rapidly increasing. Therefore, research on plant-growth-promoting bacteria (PGPB) with heavy metals tolerance has attracted increasingly more attention [18–22]. In a previous study, Efe [23] isolated nine bacterial strains with different plant-growth-promoting characteristics, such as secretion of indole-3-acetic acid (IAA), production of iron carriers, nitrogen fixation, and phosphorus solubilization, from the soil of lead–zinc mining area, and evaluated their resistance to heavy metals such as lead, zinc, and copper. In another study, Mesa-Marín et al. [24] investigated the heavy metals remediation effects of PGPB-assisted Streptomyces branching, and found that heavy metals resistant PGPB could improve Streptomyces branching and increase the potential of heavy metals remediation. On the one hand, PGPB have the functions of dissolving phosphorus and potassium as well as producing plant auxin and iron carrier, which support the survival and growth of heavy metals resistant plants in barren soil [25, 26]. On the other hand, PGPB alleviate plant oxidative stress by enhancing the synthesis of enzymatic and non-enzymatic antioxidants, helping plants to withstand external environmental pressure, and increasing the resistance of plants to heavy metals [27, 28] In addition, some heavy metals resistant PGPB can directly act on soil heavy metals, reducing heavy metals to form heavy metal complexes or producing metabolites to decrease the toxicity of heavy metals [29, 30]. Thus, heavy metals resistant PGPB play an extremely important role in microorganisms–plants combined remediation of heavy metals polluted environments.

In this study, a bacterial strain showing good lead tolerance and adsorption was screened and identified. The identified strain, *Bacillus cereus* SEM-15, showed remarkable ability to secrete IAA and dissolve inorganic phosphorus, and had good antagonistic effects against *Fusarium* and other disease-causing strains [31]. It was showed that the strain had good application potential in heavy metal lead adsorption and plant growth promotion. However, the mechanism of its action was still unclear. Together with the previous literatures, we speculated that the lead adsorption of the strain may be due to the interaction between the cell surface secretion or other substances and lead ions, and some genes related to heavy metal tolerance, adsorption and other functions may also play a certain role. Therefore, based on this assumption, the factors influencing lead adsorption efficiency of the strain were analyzed, and the surface morphology changes and possible lead adsorption mechanisms of the strain were examined by scanning electron microscopy, energy spectrum analysis, X-ray photoelectron spectroscopy, and other technologies. The functional gene information for the strain and molecular basis of heavy metal tolerance and plant growth promotion were clarified by whole genome sequencing. The results of this study provide a reference for comprehensive development and utilization of heavy metals resistant PGPB, plant growth promotion and disease prevention.

## Results

### Strains screening

#### Analysis of lead adsorption rate of the strains

**Fig. 1:**
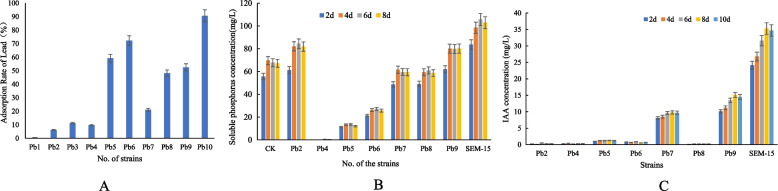
Functional screening of **A** lead adsorption, **B** inorganic phosphorus dissolution, and **C** indole-3-acetic acid (IAA) secretion of different bacterial strains. **A** Analysis of lead adsorption rate of 10 different bacterial strains in NB medium containing 150 mg/L Pb^2+^ for 12 h; **B** Analysis of inorganic phosphate dissolution ability of 10 different bacterial strains in inorganic phosphorus medium on days 2, 4, 6, and 8 of fermentation; **C** Analysis of IAA secretion ability of 10 different bacterial strains in NB medium supplemented with tryptophan on days 2, 4, 6, 8, and10 of fermentation

After gradient dilution of the pond mud, 10 bacterial strains tolerant to 300 mg/L Pb^2+^ were selected and numbered as Pb1–10. Atomic adsorption spectrometry was used to determine the residual Pb^2+^ concentration in the culture supernatant of each strain cultivated in NB medium containing150 mg/L Pb^2+^ for 12 h, and the results are shown in Fig. 1A. The lead adsorption rate of the 10 bacterial strains significantly varied, and strain Pb10 exhibited the highest adsorption rate, which reached more than 90%. The strain Pb10 was named SEM-15.

#### Analysis of inorganic phosphorus dissolution ability of the strains

By using calcium phosphate as the sole phosphorus source, the soluble phosphorus concentration in the supernatant of the fermentation broth of strains Pb2, Pb4–9, and SEM-15 was determined (Fig. 1B). Except for strain Pb4, all the tested lead-resistant strains, including SEM-15, could dissolve calcium phosphate. Among them, strains SEM-15, Pb9, and Pb2 exhibited the strongest inorganic phosphorus dissolution ability, reaching a peak on days 4–8 of detection, with soluble phosphorus concentration in the fermentation broth reaching 105.6, 84.4, and 80.2 mg/L, respectively.

#### Analysis of indole-3-acetic acid secretion ability of the strains

The strains Pb2, Pb4–9, and SEM-15 were cultured in NB medium supplemented with tryptophan, and their indole-3-acetic acid (IAA) secretion ability at different fermentation times was determined (Fig. 1C). The results revealed that strains Pb7, Pb9, and SEM-15 had the ability to secrete IAA, with SEM-15 presenting the highest IAA concentration in the supernatant of the fermentation broth (35.3 mg/L IAA on day 8 of fermentation).

### Characteristics of lead adsorption by strain SEM-15

**Fig. 2:**
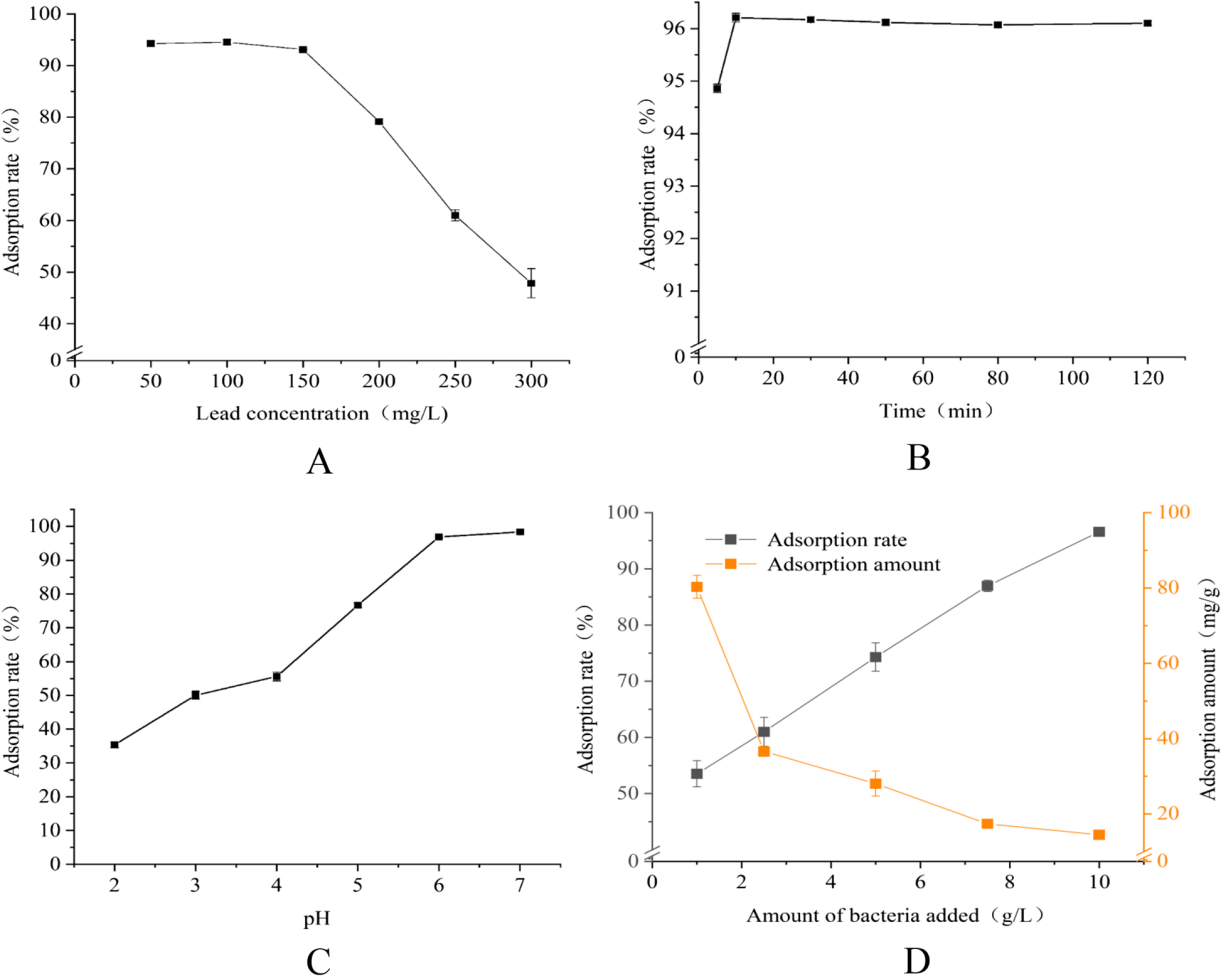
Effect of different factors on lead adsorption rate of strain SEM-15. Effect of **A** initial Pb.^2+^ concentration, **B** adsorption time, **C** pH, and **D** initial inoculum amount on the lead adsorption rate of strain SEM-15

Figure 2A shows the results of the effect of initial Pb^2+^ concentration on the lead adsorption rate of strain SEM-15. When the initial Pb^2+^ concentration was in the range of 50–150 mg/L, the lead adsorption rate of strain SEM-15 was more than 93%; however, when the initial Pb^2+^ concentration was in the range of 150–300 mg/L, the lead adsorption rate rapidly decreased, reaching 47.82% at 300 mg/L Pb^2+^. This decrease in the lead adsorption rate with the increase in the initial Pb^2+^ concentration may be owing to the competition of adsorption sites; under the condition of low Pb^2+^ concentration, the number of available adsorption sites were higher than the Pb^2+^ content, and the lead adsorption rate was relatively stable. Therefore, 150 mg/L was employed as the initial Pb^2+^ concentration in the subsequent adsorption experiments. Figure 2B shows the results of the effect of reaction time on the lead adsorption rate of strain SEM-15. The lead adsorption rate of strain SEM-15 was high, reaching more than 90% in 5 min of reaction time, and the adsorption peak reached 96.21% at 10 min. After 10 min, the lead adsorption rate of the strain remained stable between 96.07% and 96.21%. Figure 2C illustrates the effect of pH on the lead adsorption rate of strain SEM-15. The lead adsorption rate of strain SEM-15 increased with the increase in pH value. The lead adsorption rate rapidly increased at pH 2–6, and became relatively stable at pH 6–7. Figure 2D presents the effect of initial inoculum amount on the lead adsorption rate of strain SEM-15. While the lead adsorption rate of strain SEM-15 increased with the increase in the initial inoculum amount, the unit lead adsorption capacity (lead adsorption rate per bacterial cell) decreased with the increasing initial inoculum amount. An initial inoculum amount of 10 g/L produced the highest lead adsorption rate (96.58%); however, the unit lead adsorption capacity decreased to a minimum of 14.49 mg/g. In contrast, when the initial inoculum amount was 1 g/L, the lead adsorption rate was the lowest (53.52%), but the unit adsorption capacity was the highest (80.28 mg/g). An initial inoculum amount of 2.5 mg/g resulted in a balance point between the lead adsorption rate (60.98%) and unit adsorption capacity (36.59 mg/g).

### Lead adsorption mechanism of strain SEM-15

#### Scanning electron microscopy and energy spectrum analysis

**Fig. 3:**
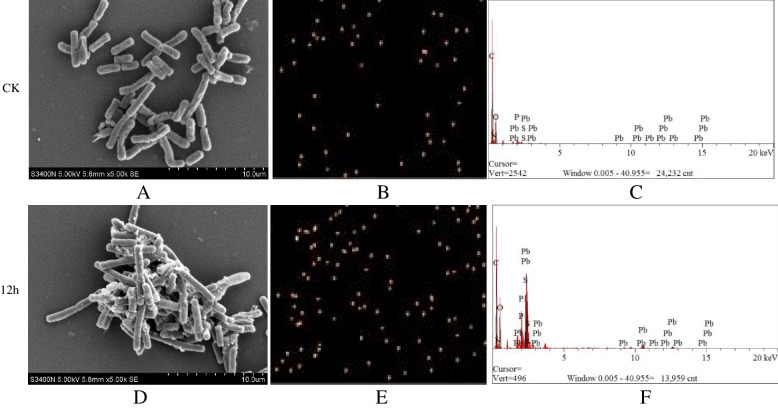
Scanning electron microscopic observation and energy spectrum analysis of strain SEM-15 before and after lead adsorption. **A** Scanning electron microscopic observation of strain SEM-15 before lead adsorption; **B** Energy spectrum analysis of the distribution of elemental lead on strain SEM-15 before lead adsorption; **C** Relevant content of elemental lead in the scanning area of strain SEM-15 before lead adsorption; **D** Scanning electron microscopic observation of strain SEM-15 after lead adsorption; **E** Energy spectrum analysis of the distribution of elemental lead on strain SEM-15 after lead adsorption; **F** Relevant content of elemental lead in the scanning area of strain SEM-15 after lead adsorption

The changes in the characteristics of surface morphology and structure of strain SEM-15 after lead adsorption were observed and analyzed by scanning electron microscopy and energy spectrum analysis. Obvious morphological changes were noted in strain SEM-15 after lead adsorption. Before lead adsorption, the cells were short rod-shaped with smooth surface (Fig. 3A). However, after lead adsorption, a large number of particles adhered to the cell surface (Fig. 3D). Energy spectrum analysis revealed dispersion of elemental lead in the scanning area (Fig. 3B, E), with more elemental lead content found in the scanning area after adsorption (Fig. 3C, F), which indicated the interaction of elemental lead with the bacterial cell surface components and subsequent aggregation on the cell surface in a granular form.

#### X-ray photoelectron spectroscopy

**Fig. 4:**
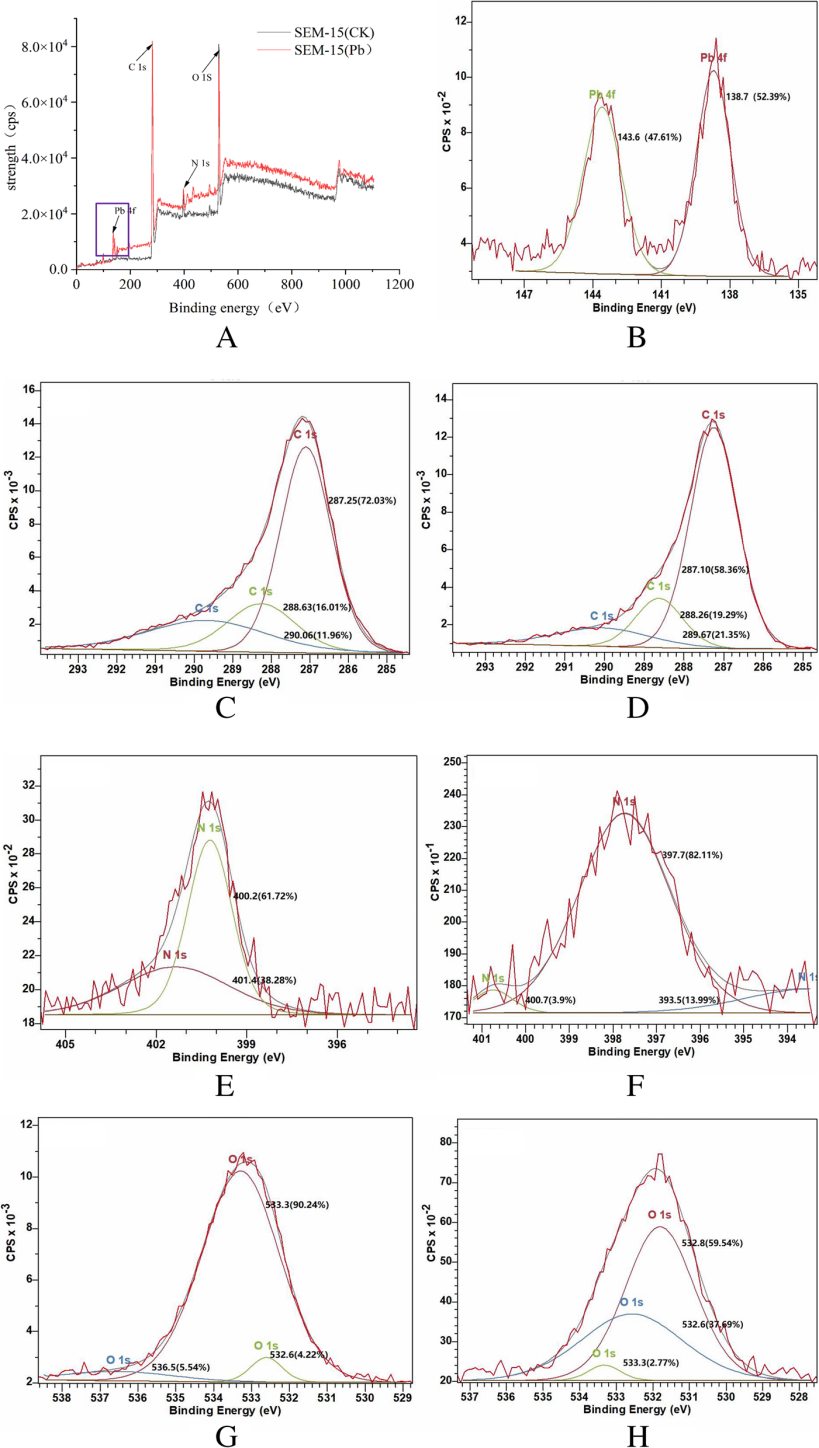
X-ray photoelectron spectroscopy analysis of strain SEM-15 before and after lead adsorption. **A** X-ray photoelectron spectroscopy of strain SEM-15 before and after lead adsorption; **B** Two Pb 4f characteristic peaks after lead adsorption; **C**, **D** Changes in binding energies of C characteristic peaks (**C**) before and (**D**) after lead adsorption; **E**, **F** Changes in binding energies of N characteristic peaks (**E**) before and (**F**) after lead adsorption; **G**, **H** Changes in binding energies of O characteristic peaks (**G**) before and (**H**) after lead adsorption

To further analyze the changes in the bacterial surface elements before and after lead adsorption, X-ray photoelectron spectroscopy was employed, and the results are shown in Fig. 4A. After lead adsorption by strain SEM-15, two new characteristic peaks appeared (Fig. 4B) with binding energies of 138.7 and 143.6 eV, both which could be attributed to Pb 4f, and the corresponding chemical bonds were Pb–O and Pb–O-R (R = functional group), respectively. In addition, the characteristic peaks of binding energies of C, N, and O also shifted to form new characteristic peaks. Table 2 lists the major binding energy peak changes and proportions. Three C 1 s characteristic peaks with binding energies of 287.25, 288.63, and 290.06 eV were detected (Fig. 4C, D), and their peak area ratios significantly changed before and after lead adsorption (Table 1). While two characteristic peaks of N 1 s with binding energies of 400.2 and 401.4 eV were observed before lead adsorption (Fig. 4E), the characteristic peaks shifted after lead adsorption with binding energies of 393.5, 397.7, and 400.7 eV (Fig. 4F). Furthermore, before lead adsorption, three characteristic peaks of O 1 s were detected, with binding energies of 532.6, 533.3, and 536.5 eV (Fig. 4G); however, after lead adsorption, the three characteristic peaks shifted with binding energies of 532.6, 532.8, and 533.3 eV (Fig. 4H). These findings revealed that the N-, O-, and C-containing functional groups on the bacterial cell surface participate in the lead adsorption process, with lead deposition on the bacterial cell surface affecting the binding energy of functional groups, causing a shift in their characteristic peaks.

**Table 1:** Changes in the binding energy peaks of Pb, C, N, and O on the surface of strain SEM-15 before and after lead adsorption

	Binding Energy Peak		Proportion Statistics(%)	
	No	Binding Energy(eV)	CK	Pb^2+^ adsorption
Pb	1	138.7	/	52.39
	2	143.6	/	47.61
C	1	287.25	72.03	58.36
	2	288.63	16.01	19.29
	3	290.06	11.96	21.35
N	1	393.5	/	13.99
	2	397.7	/	82.11
	3	400.2	61.72	/
	4	401.4	38.28	/
	5	400.7	/	3.9
O	1	532.6	4.22	37.69
	2	532.8	/	59.54
	3	533.3	90.24	2.77
	4	536.5	5.54	/

#### Fourier transform infrared spectroscopy

**Fig. 5:**
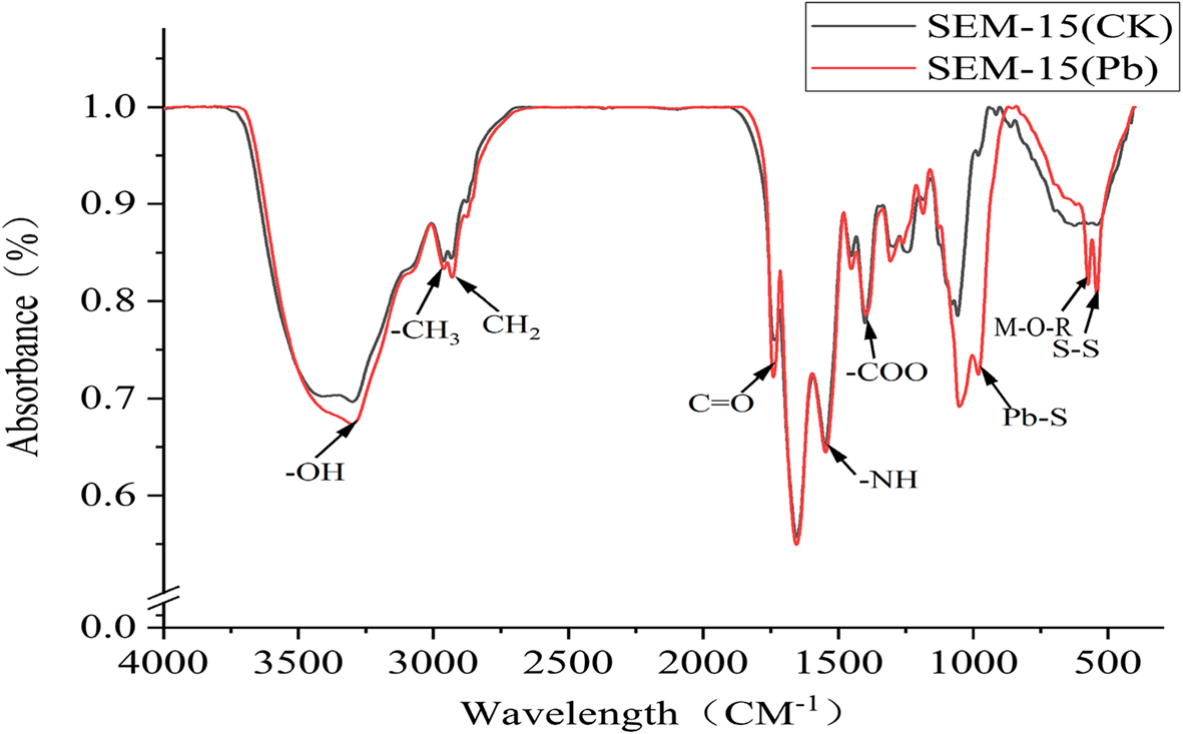
Fourier transform infrared spectroscopy analysis of changes in the functional groups on the bacterial cell surface before and after lead adsorption. Note: Arrows show the Fourier transform infrared spectroscopy spectra adsorption peaks of different functional groups

To further analyze the functional groups involved in lead adsorption by strain SEM-15, Fourier transform infrared spectroscopy was used to detect the changes in the functional groups on the bacterial cell surface before and after lead adsorption (Fig. 5). Comparison of the Fourier transform infrared spectroscopy spectra before and after lead adsorption revealed some changes in the shape of the characteristic peaks before and after lead adsorption (Table S1). In particular, three new vibration adsorption peaks were formed at 573.91, 541.68, and 980.24 cm^−1^ after lead adsorption, which corresponded to the vibration adsorption peaks of M–O and O-M–O (M = metal ions), S–S, and Pb–S bonds, respectively. In contrast, three vibration adsorption peaks at 623.90, 861.27, and 914.74 cm^−1^ disappeared after lead adsorption, which corresponded to the vibration adsorption peaks of alkyn CH bending (out-of-plane), benzene ring C-H out-of-plane deformation, and aliphatic ether C–O–C symmetric pull-up, respectively. These newly formed or disappeared functional groups after lead adsorption indicated the involvement of redox reactions in the process of lead adsorption. Besides, the characteristic adsorption peaks representing functional groups, such as N–H and O–H of protein amide, CH_3_ of fatty acids, CH_2_ of lipids, etc., also presented a certain shift, implying that these functional groups may also be involved in the process of lead adsorption.

### Whole genome sequencing and bioinformatics analysis

**Fig. 6:**
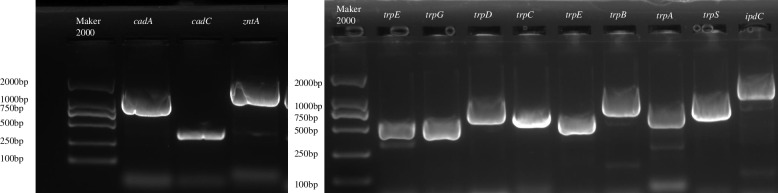
PCR validation of some annotated genes of strain SEM-15

By using BGI independent sequencing platform, the whole genome of strain SEM-15 was sequenced. The genome analysis results showed that the full-length genome of strain SEM-15 was 6,131,470 bp, including one chromosome sequence and four plasmid sequences, with a total of five contigs; the chromosome sequences were 5,259,723 bp in length, while the four plasmid sequences were 537,825, 265,959, 54,444, and 13,519 bp in length, respectively, with an average GC content of 34.94%. A total of 6151 genes were annotated in the genome, of which 5956 were protein-coding sequences (CDSs). Moreover, 42 rRNA-, 107 tRNA-, and 46 ncRNA-coding genes were further predicted, and 13 complete prophage regions were identified, of which 11 were located in chromosome1 and one was located in Plasmid3 and Plasmid4. All the sequence data were assembled. Figure 6 shows the draft whole genome of strain SEM-15, and Table 2 lists the genome sequence characteristics of strain SEM-15.

**Table 2:** Genomic characteristics of strain SEM-15

Features	Numerical value
Genome size(bp)	6 131 470
Chromosome size(bp)	5 259 723
Plasmid 1 size(bp)	537 825
Plasmid 2 size(bp)	265 959
Plasmid 3 size(bp)	54 444
Plasmid 4 size(bp)	13 519
Contigs	5
Average content of G + C(%)	34.94%
Gene number	6 151
RNA number	195
rRNA number	14,14,14(5S,16S,23S)
tRNA number	107
ncRNA number	46
Number of prophages	13
CRISPR number	1

### Analysis of heavy metals resistance and plant growth promotion related genes of strain SEM-15

Analysis of the genomic data revealed that the genes involved in heavy metals (including lead, arsenic, cadmium, zinc, copper, etc.) resistance were annotated in the genomic sequence of the strain SEM-15 (Table 3), suggesting that the strain may have multiple heavy metals resistance. Besides, some genes.

**Table 3:**
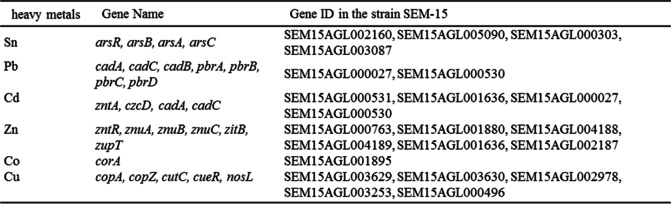
Genes related to heavy metals tolerance in the genome of strain SEM-15

involved in IAA synthesis and secretion, phosphorus and potassium dissolution, etc., including a variety of phosphatase, transporter, potassium ion transporter, and other related genes, were also found in the genome of strain SEM-15 (Table 4), indicating the molecular basis for IAA secretion and phosphorus and potassium dissolution ability of strain SEM-15. Subsequently, 12 genes involved in lead resistance, IAA synthesis and secretion, etc., were randomly selected for PCR verification, and the results revealed that the size of the PCR product of the gene was consistent with the predicted value (Fig. 6).

**Table 4:**
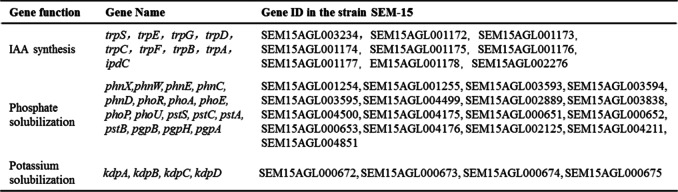
Genes related to plant growth promotion in the genome of strain SEM-15

## Discussions

### Functional analysis of strain SEM-15

Although some PGPB could indirectly promote phytoremediation of heavy metals via IAA production [32], phosphorus solubilization, potassium solubilization [33], siderophore production [34–36], ACC deaminase activity, nitrogen fixation, etc. [37], the growth-promoting characteristics and heavy metals remediation ability can significantly vary among different PGPB [38–40]. In a previous study, nine lead-resistant bacterial strains were isolated, among which *B. cereus* DE-1 exhibited the ability to secrete IAA, but did not present other growth-promoting functions [23]. Ortiz-Ojeda et al. [38] screened some bacteria with lead and cadmium resistance, and observed that these bacteria had good ability to secrete IAA. Ma et al. [39] found that *Bacillus* sp. SC2b could tolerate a maximum of 1400 mg/L Pb^2+^, along with functions of phosphate solubilization (56.6 mg/L), IAA secretion (64.8 mg/L), and iron carrier functions. In the present study, the strain SEM-15 exhibited good lead adsorption performance. At 150 mg/L Pb^2+^, strain SEM-15 exhibited more than a 90% lead adsorption rate and good ability to dissolve inorganic phosphorus and secrete IAA. These results showed that *B. cereus* SEM-15 has good application potential in plant growth promotion and lead pollution remediation, especially in plants–microorganisms combined remediation.

### Lead adsorption characteristics of strain SEM-15

The adsorption of heavy metals by microbial strains was affected by many factors, such as temperature, pH, biomass concentration, etc. [13, 41]. Cho and Kim [13] found that the ability to remove Pb^2+^ from aqueous solution of the yeast *Rhodotorula glutinis* was affected by the contact time, initial pH, temperature and biomass concentration. Sari and Tuzen [41] investigated the biosorption characteristics of Pb(II) and Cd(II) ions from aqueous solution using the macrofungus (*Amanita rubescens*). In the present study, the lead adsorption characteristics of strain SEM-15 in inorganic salt solution was analyzed. It was found that the lead adsorption rate reached a peak within 10 min of contact. Under nutrient-free conditions, the biosorption of bacterial strains is dominated by in vitro adsorption, which is fast and efficient [42]. One of the important factors affecting adsorption of metal ions is pH of the solution. This factor directly related with competition ability of hydrogen ions with metal ions to active sites on the stain surface [41, 43]. The increase in pH can promote lead adsorption by the strain SEM-15, and the best lead adsorption efficiency has been observed at pH 6–7. Furthermore, the biomass dosage determined the adsorption capacity for a given initial concentration. The increase in the initial inoculum amount improved the lead adsorption rate of the strain SEM-15. We speculated that with the increase in the initial inoculum amount, the adsorption sites on the bacterial surface increased and more Pb^2+^ could come into contact and combine, resulting in significant increase in lead adsorption rate. In contrast, Pb^2+^ concentration was negatively correlated with lead adsorption, which may be owing to the competition of adsorption sites. It must be noted that under the condition of low Pb^2+^ concentration, the number of lead adsorption sites is higher than the Pb^2+^ content and the adsorption rate is relatively stable; however, when the adsorption site reaches a critical value, the Pb^2+^ content becomes sufficient and the lead adsorption rate decreases [44]. The adsorption efficiency of heavy lead metal could be related to the ratio of the initial Pb^2+^ concentration to the biomass dosage [45, 46].

### Lead adsorption mechanism of strain SEM-15

The main mechanism of detoxification for Pb2 + included biosorption, bioaccumulation, complexation, and biomineralization etc. [47]. Scanning electron microscopic observation of the changes in the cell surface before and after lead adsorption by strain SEM-15 revealed that the morphology and structure of the cells changed after lead adsorption, with cell surface covered with fine particles. Energy spectrum analysis showed that the Pb^2+^ content on the surface of the cell significantly increased after lead adsorption. We speculated that it may be the biosorption between the strain surface and Lead ions. Shin [48] found that endophytic *Bacillus* sp. MN3-4 exhibited more extracellular Pb^2+^ adsorption and sequestration by using scanning electron microscopy–energy dispersive X-ray spectroscopy. Furthermore, X-ray diffraction and transmission electron microscopy studies revealed that the Pb^2+^ on the bacterial surface were gradually transformed into nanocrystals [49].

The surface functional groups of the strain may involve in the biosorption process of heavy metal ions [50]. To investigate the changes in Pb^2+^ valence and functional groups on the bacterial cell surface after lead adsorption by strain SEM-15, X-ray photoelectron spectroscopy and Fourier transform infrared spectroscopy were used to study the variations in the energy spectrum before and after lead adsorption. The X-ray photoelectron spectroscopy results showed shifts in the characteristic binding energy peaks of C, N, and O before and after lead adsorption by the strain SEM-15, generating new characteristic peaks. Similarly, Fourier transform infrared spectroscopy analysis also demonstrated that the peaks of N–H, O–H, -CH_3_, -CH_2_, C-N, COO-, PO_2_, C–O–C, and P-O-C functional groups significantly shifted, indicating that N-, O-, and C-containing functional groups may be involved in the lead adsorption process. The Fourier transform infrared spectroscopy spectra showed the formation of two new vibration adsorption peaks at 541.68 and 980.24 cm^−1^ after lead adsorption, which corresponded to S–S and Pb–S bonds, respectively, thus implying the formation of disulfide and Pb–S bonds in the lead adsorption process. Govarthanan et al. [51] reported that *Bacillus* KK1 screened from mine tailings can convert carcinogenic and toxic Pb(NO_3_)_2_ into low-toxic PbS or normal non-toxic PbSiO_3._ The energy spectrum results of the present study showed two new characteristic peaks with binding energy of 138.7 and 143.6 eV (both attributed to Pb 4f) after lead adsorption by strain SEM-15, which corresponded to Pb–O and Pb–O-R (R = functional group) bonds, respectively. In addition, the Fourier transform infrared spectroscopy also revealed the appearance of a new vibration adsorption peak at 573.91 cm^−1^ after lead adsorption, which corresponded to M–O and O-M–O (M = metal ion) bonds, demonstrating that the X-ray photoelectron spectroscopy results were consistent with the Fourier transform infrared spectroscopy findings, and that the process of lead adsorption and passivation by strain SEM-15 involved the formation of Pb–O and Pb–O-R bonds; however, the specific occurrence process requires further investigation.

### Genetic analysis of heavy metals tolerance of strain SEM-15

A previous study noted that strain SEM-15 not only exhibited good resistance to lead and lead adsorption capacity, but also showed good resistance to other heavy metals such as arsenic, cadmium, zinc, and copper [31]. In the present study, to better analyze the heavy metals tolerance and growth-promoting mechanism of strain SEM-15, the whole genome of the strain was sequenced and analyzed. The genome of strain SEM-15 was found to contain genes related to tolerance of heavy metals such as arsenic, cadmium, zinc, copper, and lead, IAA synthesis and secretion, and phosphate and potassium solubilization.

The microbial resistance to arsenic is known to originate from the *ars* operon containing five genes, *arsA*, *arsD*, *arsB*, *arsC,* and *arsR* [52], and strain SEM-15 was found to contain most of the genes of the *ars* operon (*arsA*, *arsB*, *arsC,* and *arsR*), indicating its potential for arsenic tolerance [53]. A co-mediated expression regulation system (czc system) is known to regulate the microbial resistance to cobalt, zinc, and cadmium, and the genome of strain SEM-15 was noted to contain cadmium ion efflux pump genes *cadA*, *cadC*, *czcD* of the czc efflux system and *corA* gene of the cor system. The microbial resistance to copper is mainly mediated by the *cop* operon (*copA*, *copB*, *copY,* and *copZ*) [54, 55], and the genome of strain SEM-15 contained *copA*, *copZ*, *cutC,* and other operon genes. Microbial resistance to lead is mainly regulated by the efflux pump genes *cadA*, *cadB,* and *cadC* as well as the *pbr* system (*pbrA*, *pbrB*, *pbrC,* and *pbrD*) [56], and the genome of strain SEM-15 contained *cadA*, *cadB,* and *cadC* genes, but not the pbr system genes. It must be noted that the heavy metals tolerance mechanism of bacteria exhibits a certain unity and comprehensiveness, and genes involved in other heavy metals tolerance and drug tolerance can directly or indirectly cause lead tolerance in strains. For example, genes related to arsenic resistance can reduce lead toxicity by increasing phosphate metabolism and subsequently forming a certain tolerance to lead [57, 58]. Furthermore, the cadmium ion efflux pump genes, *cadA* and *cadC,* also play a role in the efflux of Pb^2+^, while P-type ATPase genes, such as *zntA*, *zntR*, *actP* and *czcD* genes, are also involved in the transport of Pb^2+^ via cell membrane [59]. In the present study, analysis of the annotated genes of the genome of strain SEM-15 revealed the presence of a variety of heavy metals resistance genes, which may constitute the molecular basis of the resistance of strain SEM-15 to various heavy metals.

## Conclusions

*B. cereus* SEM-15 showed strong ability to dissolve inorganic phosphorus, secrete acidindole-3-acetic acid and absorb the heavy metal Lead. The optimal conditions for heavy metal lead adsorption by *B. cereus* SEM-15 (adsorption time, initial lead ion concentration, pH, and inoculum amount were 10 min, 50–150 mg/L, 6–7, and 5 g/L, respectively) in nutrient-free environment. The functional groups on the *B. cereus* SEM-15 cell surface involved adsorption process, such as Pb–O, Pb–O-R, and Pb–S bonds etc. The genes related to heavy metals tolerance and plant growth promotion was found in the genome of *B. cereus* SEM-15. The results obtained help to clarify the mechanism of heavy metals resistance of the strain, and provide a reference for further research on plants–microorganisms combined remediation of heavy metals pollution using *B. cereus* SEM-15.

## Materials and methods

### Medium formulation

Inorganic phosphorus medium contained the following (g/L): glucose, 10.0; yeast extract, 0.5; Ca_3_(PO_4_)_2_, 5.0; (NH_4_)_2_SO_4_, 0.5; KCl, 0.2; MgSO_4_, 0.1; MnSO_4_, 0.0001; and FeSO_4_, 0.0001.

IAA medium comprised the following (g/L): beef extract, 3.0; peptone, 5.0; NaCl, 5.0; and tryptophan, 1.0.

### Screening of strains with lead-adsorption ability

A total of 2 g of pond soil sample were collected, ground in a mortar, and mixed evenly. The obtained supernatant was collected and subjected to gradient dilution, and then inoculated onto screening medium (NA medium) supplemented with 300 mg/L Pb^2+^, and incubated for 48 h at 37 °C. Subsequently, the colonies grown on the plate were picked and streaked to obtain single colonies, and strains with volume ratio of 20% glycerin were stored in a refrigerator at − 80 °C.

Selected single colony of the strains and inoculated it into 5 ml NB medium, cultured at 37 °C and 180 r/min for overnight. The activated strains were inoculated into NB medium containing 150 mg/L Pb^2+^ in the proportion of 1% by volume, and incubated in a rotary shaker at 37 °C and 180 r/min. After 12 h of incubation, samples were collected and centrifuged, and the concentration of soluble Pb^2+^ in the supernatant was determined by atomic adsorption spectrometry [31, 60].

### Determination of strains with ability to dissolve inorganic phosphorus during fermentation

The isolates were inoculated into 100 mL of inorganic phosphorus medium containing calcium phosphate as the sole phosphorus source, and incubated in a rotary shaker at 200 r/min and 30 °C. Samples were collected between 2 and 8 days, and the cells were isolated by centrifugation at 83.3 × g for 10 min. The supernatant obtained was used to detect the concentration of soluble phosphorus by the molybdenum-antimony anti-colorimetric method [61]. *Bacillus megaterium* GW-1–0201-1305–04 (obtained from Weiyuan Biological Technology Co., Ltd. Guangzhou, China) was employed as the positive control and cultivated under the same conditions.

### Determination of IAA secretion by the strains during fermentation

Individual isolates were inoculated into NB medium containing tryptophan (100 mg/L) at a proportion of 1% and cultured at 37 °C and 180 r/min, and sampling was commenced after 2 days. Subsequently, the fermentation broth was centrifuged at 5876 × g for 10 min, and 2 mL of the supernatant were mixed with 50 μL of 83% orthophosphoric acid and 4 mL of Salkowski reagent [62, 63]. Formation of a pink solution indicated IAA production (observed until 10 days). The concentration of IAA produced by the strains was determined by colorimetric method, and the absorbance value (OD_535_) of the reaction solution was determined, with sterile NA medium containing tryptophan as the control.

### Analysis of factors affecting lead adsorption by the strains

#### Effect of time on lead adsorption efficiency of the strains

The activated broth of strains cultured for 12 h was mixed with 150 mg/L Pb^2+^ solution at an inoculum concentration of 5%, sealed with a sealing film, and incubated at 35 °C and 180 r/min. Subsequently, samples were collected in triplicates at 5, 10, 30, 50, 80, and 120 min, respectively, and the Pb^2+^ content in the supernatant was determined by atomic adsorption spectrometry, with lead solution as control.

#### Effect of pH on lead adsorption efficiency of the strains

The activated broth of strains cultured for 12 h was mixed with 150 mg/L Pb^2+^ solution at different pH (pH from 2 to 7, respectively) at an inoculum concentration of 5%, sealed with parafilm, and cultured at 35 °C and 180 r/min. Each pH gradient was examined in triplicate, and samples were collected after 120 min. The Pb^2+^ content in the samples was determined by atomic adsorption spectrometry, with lead solution as the control.

#### Effect of initial Pb^2+^ concentration on lead adsorption efficiency of the strains

The activated broth of strains cultured for 12 h was mixed with 50, 100, 150, 200, 250, and 300 mg/L Pb^2+^ solution, respectively, at an inoculum concentration of 5%, sealed with parafilm, and incubated at 35 °C and 180 r/min. The experiment was performed in triplicate and samples were collected at 120 min. The Pb^2+^ concentration in the samples was determined by atomic adsorption spectrometry, with lead solution as control.

#### Effect of inoculum concentration on lead adsorption efficiency of the strains

The activated broth of strains cultured for 12 h was mixed with 150 mg/L Pb^2+^ solution at an inoculum concentration of 1%, 2.5%, 5%, 7.5%, and 10%, respectively, sealed with parafilm, and incubated at 35 °C and 180 r/min. The experiment was performed in triplicate and samples were collected at 120 min. The Pb^2+^ concentration in the samples were ascertained by atomic adsorption spectrometry, with lead solution as the control.

### Scanning electron microscopy

For scanning electron microscopy analysis, the strains were cultured in NA medium containing 150 mg/L Pb^2+^ for 12 and 24 h, respectively. The control comprised NA medium without Pb^2+^. A total of 10 mL of the culture broth were centrifuged at 2292 × g for 10 min, washed with sterile water, and centrifuged again at 5000 rpm for 10 min to collect the cells. The cells were suspended in 40-fold volume of 2.5% glutaraldehyde fixative, stored in a refrigerator for more than 24 h at 4 °C, rinsed with phosphate buffer for 2–3 times, and subjected to gradient dehydration with 50%, 70%, 90%, and 100% ethanol, respectively. After dehydration, the cells were treated with ethanol-tert-butanol solution (V1:V2 = 1:1) for 20 min, and with 100% tert-butanol twice for 20 min each. Subsequently, the samples were freeze-dried, sputter-coated using ion sputtering equipment (IXRF SYSTEMS, MSP-2S, Japan), and observed and photographed under a scanning electron microscope (Hitachi, S3400N, Japan).

### X-ray photoelectron spectroscopy

For X-ray photoelectron spectroscopy, the strains were cultured in NA medium containing 150 mg/L Pb^2+^ for 24 h. The control included NA medium without Pb^2+^. After 24 h, 50 mL of the culture broth were centrifuged at 2292 × g for 10 min and washed with sterile water. After vacuum freeze-drying (Tokyo Rika, FDU-2110, Japan) for 48 h, 100.00 mg of KBr powder dried to constant weight was added, mixed in a mortar, and pressed into a transparent sheet using a tablet machine. The cell surface elements were excited using an X-ray photoelectron spectrometer (Kratos, Axis Ulra DLD, England), and the photoelectron spectrum data were recorded using an energy analyzer.

### Fourier transform infrared spectroscopy analysis

For Fourier transform infrared spectroscopy analysis, the samples preparation procedure was similar to that employed for X-ray photoelectron spectroscopy. The thin sample slices were scanned using Fourier transform infrared spectrometer in the range of 4000–500 cm^−1^ for bacteria, and the spectral results were recorded.

### Genome sequencing and bioinformatics analysis

The genomic DNA was extracted from the strains cultured in NB medium using miniBEST Bacteria Genomic DNA Extraction Kit (TaKaRa code DV810A; TaKaRa, DaLian, China), according to the manufacturer’s instructions, and sent to BGI (Wuhan, China) for quality inspection and genome sequencing. The whole genome sequencing and sequence assembly analysis were performed as described previously [64].

The raw data were filtered to remove adapters, such as low-quality reads, to generate clean data. The Short Oligonucleotide Analysis Package (SOAPdenovo) software (www.soap.genomics.org.cn) was used to assemble reads after filtering and perform bioinformatic analysis, including genomic component analysis, comparative genomic analysis, and gene function analysis.

Functional prediction for rRNA, tRNA, and sRNA was performed using the software Glimmer [65], RNAmmer [66], tRNAscan [67], Infernal, and Rfam [68]. The software Tandem Repeat Finder [69] was used to predict series repeat sequences, small satellites, and microsatellite sequences; PhiSpy software [70] was employed to predict the prophage; and CRISPRCas Finder software [71] was utilized to identify CRISPRs, etc.

### Declaration

The genomic sequence assembly and analysis data of the strain SEM-15 was submitted to the GenBank database under submission number CP095377–CP095381.

### PCR verification of related genes

The primers were designed using the software Primer Premier 5 according to the sequence of the genes, including some lead tolerance, IAA production, and phosphorus solubilization related genes, of strain SEM-15. All the primers were synthesized by Beijing Liuhe Huada Gene Technology Co., Ltd, and the relevant primer sequences are shown in Table 5.

**Table 5:**
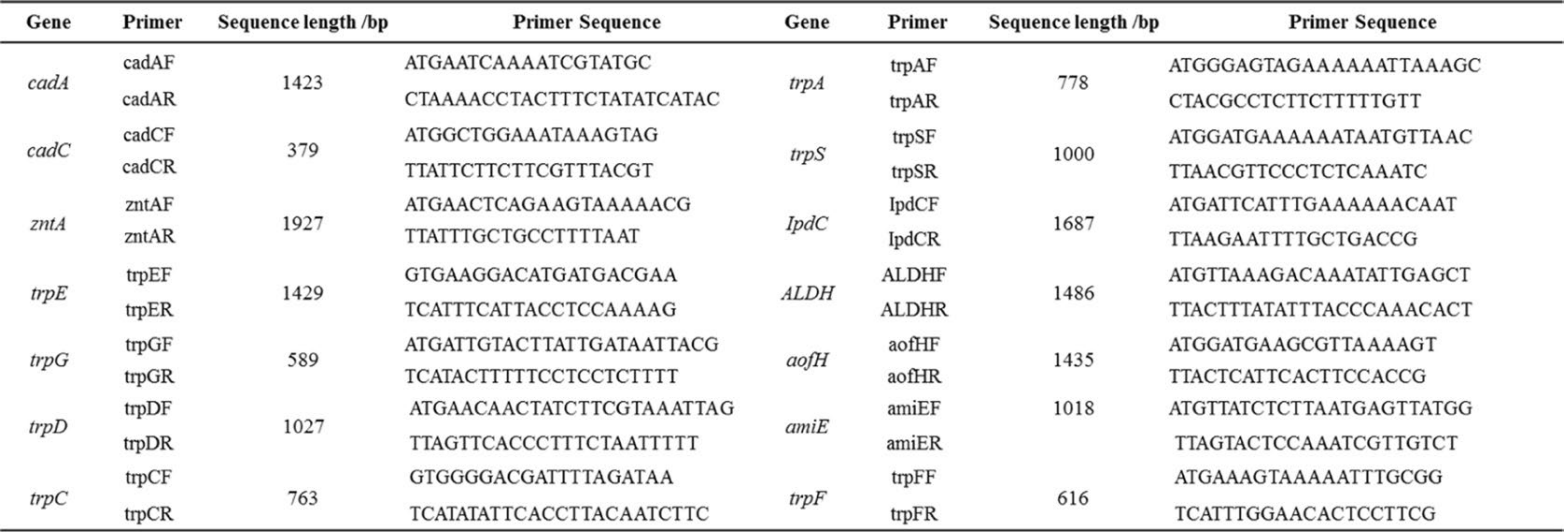
PCR primer sequences of related genes

The genomic DNA of strain SEM-15 was extracted using the bacterial genomic DNA purification kit (Beijing Transgene), according to the manufacturer’s instructions. PCR amplification was performed using genomic DNA as template. The 50-µL reaction system for PCR comprised the following: 25 µL of 2 × EasyTaq® PCR SuperMix, 1.5 µL each of forward primer and reverse primer (10 µM), 1.5 µL of DNA template, and 20.5 µL of sterile water. The PCR amplification conditions were as follows: pre-denaturation at 95 °C for 3 min; 35 cycles of denaturation at 95 °C for 30 s, annealing at 50 °C for 30 s, and extension at 72 °C for 1–2 min (this parameter was based on the actual PCR product length); and a final extension at 72 °C for 7 min.

### Statistical analysis

Comparisons of the amount of inorganic phosphorus solubilized and IAA secreted by strain SEM-15 were conducted using ANOVA.

## Supplementary Information

**Additional file 1: Table S1.** Changes in the adsorption peaks of functional groups on the surface of strain SEM-15 before and after lead adsorption.

## Data Availability

The genomic sequence assembly and analysis data of the strain SEM-15 was submitted to the GenBank database repository under submission number CP095377–CP095381.
