# Supplementary material for: Mechanism of lead adsorption by a Bacillus cereus strain with indole-3-acetic acid secretion and inorganic phosphorus dissolution functions
Source: BMC Microbiol. 2023 Mar 4;23:57. doi: 10.1186/s12866-023-02795-z (PMC9985246; doi:10.1186/s12866-023-02795-z)
Supplement: Supplementary file 1 — Additional file 1: Table S1. Changes in the adsorption peaks of functional groups on the surface of strain SEM-15 before and after lead adsorption. [file 12866_2023_2795_MOESM1_ESM.docx]

supplementary file

**Table S1 Changes in the adsorption peaks of functional groups on the surface of strain SEM-15 before and after lead adsorption**

| **Adsorption Wavelength（cm^-1^）** | | **Changes** | **Corresponding functional group changes** |
| --- | --- | --- | --- |
| **Before** | **After** |  |  |
| 3300.26 | 3301.45 | Blue shift | Amide A: N-H and O-H stretching vibration of protein |
| 2962.87 | 2961.82 | Red shift | Asymmetric stretching of fatty acid CH3 |
| 2933.66 | 2931.40 | Red shift | Asymmetric stretching of lipid CH2 |
| 1735.61 | 1741.37 | Blue shift | Stretching of triglycerides and cholesterol esters C = O |
| 1548.13 | 1546.80 | Red shift | Mainly protein amide II band N-H bending and C-N stretching |
| 1453.18 | 1452.21 | Red shift | Bending of lipid CH2 |
| 1401.04 | 1398.55 | Red shift | Symmetrically stretching of amino acid side chains and fatty acids COO |
| 1294.06 | 1306.17 | Blue shift | Protein amide III band component |
| 1243.60 | 1260.42 | Blue shift | Mainly asymmetric stretching of nucleic acids PO_2_ |
| 1184.90 | 1185.57 | Blue shift | Stretching vibration absorption peak of alcohol C-OH |
| 1080.70 | 1127.82 | Blue shift | Symmetrical stretching of nucleic acid and phospholipid PO_2_ |
| 1058.52 | 1052.30 | Red shift | Polysaccharide C-O-C, P-O-C symmetric stretching on peptidoglycan |
| NA | 980.24 | New formed | Pb-S bond |
| 914.74 | NB | Disappear | Aliphatic ether C-O-C symmetric stretching |
| 861.27 | NB | Disappear | Vibration absorption peak of out of plane deformation of benzene ring C-H |
| 623.90 | NB | Disappear | Alkyn C-H bend (out of plane) |
| NA | 573.91 | New formed | Vibration absorption peak of M-O and O-M-O（M- metal ion） |
| NA | 541.68 | New formed | Vibration absorption peak of S-S |

Note: NA: No adsorption peak at this wavelength before lead adsorption; NB: No adsorption peak at this wavelength after lead adsorption
